# Pulmonary Malformations: Predictors of Neonatal Respiratory Distress and Early Surgery

**DOI:** 10.21699/jns.v5i3.375

**Published:** 2016-07-03

**Authors:** Sara Costanzo, Claudia Filisetti, Claudio Vella, Mariangela Rustico, Paola Fontana, Gianluca Lista, Salvatore Zirpoli, Marcello Napolitano, Giovanna Riccipetitoni

**Affiliations:** 1 Department of Pediatric Surgery, V. Buzzi Children's Hospital, Milano, Italy; 2 Department of Surgery, PhD School of Experimental Medicine, University of Pavia, Italy; 3 Maternal-Fetal Department, V. Buzzi Children's Hospital, Milano, Italy; 4 Neonatal Intensive Care Unit, V. Buzzi Children's Hospital, Milano, Italy; 5 Radiology Department, V. Buzzi Children's Hospital, Milano, Italy

**Keywords:** Pulmonary malformations, Prenatal diagnosis, CPAM volume ratio, Fetal therapy, Neonatal resuscitation

## Abstract

**Objectives:** The objective of our study is to retrospectively analyze a single-centre series of antenatally detected pulmonary malformations (PM) and to evaluate their postnatal outcome.

**Materials and Methods:** We retrospectively reviewed all prenatally diagnosed PM patients referred to our Centre in the period between January 1999 and December 2014. All cases were diagnosed by one of our Maternal-Fetal Specialists by US examination. Congenital pulmonary airway malformation (CPAM) volume ratio (CVR), development of fetal complications, need for fetal therapy, need for neonatal resuscitation and timing of surgery were analyzed.

**Results:** A total of 70 fetuses were diagnosed with a PM in the period of study. An initial CVR higher than 1.6 was found in 16/70 patients (22.8%); 14/16 developed fetal complications (p less than .0001). Fifty-six fetuses (80%) did not develop any complications during pregnancy. To all complicated cases a prenatal treatment was offered, carried out in 12 (1 termination, 1 refusal). Survival rate was 100%. Sixty-three fetuses (90%) were asymptomatic at birth and did not require any neonatal resuscitation. Six patients submitted to fetal therapy and one untreated presented with neonatal respiratory distress, required mechanical ventilation at birth and early surgery in the neonatal period (7/70, 10%).

**Conclusion:** CVR > 1.6 and the presence of fetal complications can be considered as predictors of respiratory distress at birth and of the need for early surgery. Nevertheless, the vast majority of PM are asymptomatic at birth and only a small group of fetuses require prenatal and postnatal treatment and support.

## INTRODUCTION

Pulmonary malformations (PM) represent a heterogeneous group of congenital anomalies. Previously reported as rare entities, their incidence has risen from 1 in 25,000 to 1 in 2500 due to the increase in prenatal detection during routine ultrasound scanning [1]. The most common malformations of the lower respiratory tract are congenital pulmonary airways malformations (CPAM) and bronchopulmonary sequestration (BPS). Both CPAM and BPS may coexist within the lung mass as a so-called "hybrid" lesion [2]. One lesion that can be part of the differential diagnosis and has been described with increasing frequency is congenital lobar emphysema (CLE) and segmental hyperinflation [3, 4]. 

The natural history of a fetal lung mass is variable and the overall prognosis depends on the size of the lesion and on the grade of the secondary physiologic derangement caused by compression from the mass on surrounding structures [5-7].

As many as 15% of CPAM decrease in size during gestation [8] and up to 70-75% prenatally identified pulmonary lesions are asymptomatic at birth [9].

Larger masses, inversely, may lead to compression of mediastinum, vena cava and heart, hypoplasia of normal lung tissue, mediastinal shift; esophageal compression that interferes with fetal swallowing of amniotic fluid and results in polyhydramnios; pleural effusion and cardiovascular compromise leading to fetal hydrops and death [3, 5, 7]. Fetuses developing prenatal complications have a worse prognosis, particularly in the presence of hydrops, and fetal intervention is indicated [4, 6].

The ongoing improvements of prenatal imaging and the widespread use of obstetric ultrasound allow for antenatal diagnosis of smaller lesions, thus leading to a higher incidence of diagnosed PM. Subsequently, the overall prognosis of antenatally diagnosed PM has considerably improved [4, 10].

Given these premises, it is of utmost importance to establish valid tools for prenatal risk stratification, in order to identify subgroups of patients that need a closer antenatal US follow-up and that might be at increased risk for respiratory morbidity at birth.

We conducted a retrospective study on our series of PM, diagnosed, followed-up, born and treated at the same tertiary care Centre, analyzing the evolution of prenatal lesions and the need for fetal therapy. We also correlated the prenatal aspects of PM masses with the need for neonatal resuscitation and the surgical timing.

## MATERIALS AND METHODS

In this single centre retrospective cohort study, we reviewed all prenatal PM referred to the Maternal-Fetal Unit of our Centre, in the period between January 1999 and December 2014.

All cases were diagnosed by one of our Maternal-Fetal Specialists by US examination.

In each case, a measure of the CPAM volume ratio (CVR) was obtained. The CVR is a sonographic indicator that has been proposed for the evaluation of fetuses at risk for hydrops and possible intervention [8]. It is the volume of the mass normalized for gestational age. The CPAM volume is estimated using the formula for a prolate ellipse, multiplying the length, height and width of the mass by a 0.52 correction factor. The CVR is obtained by dividing the CPAM volume by the head circumference (measured in cm). As per study protocol, patients with a CVR > 1.6 underwent weekly ultrasound follow-up, while patients with a CVR ≤ 1.6 underwent a follow-up ultrasound on alternate weeks. 

The development of fetal complications (severe mediastinal shift, hydrops, or hydrothorax) and the need for fetal therapy were examined. Hydrops was defined as the abnormal accumulation of serous fluid in 2 or more fetal compartments, including ascites, pleural effusion, pericardial effusion or skin edema [5, 10].

Fetal therapies, indicated by the Maternal-Fetal Specialist in the presence of fetal complications, consisted of: thoraco-amniotic shunt (TAS), steroid administration (betamethasone 12 mg daily for two days) and alcohol injection.

The need for neonatal resuscitation, the postnatal outcome and the timing of surgery were analysed.

Statistical analysis was performed through Chi-square test and Fisher's exact test on GraphPad® online platform.

The protocol was approved by the Ethical Committee of our hospital and parental informed consent was obtained in all cases.

## RESULTS

A total of 70 fetuses were diagnosed with a PM at our Centre in the period of study. Prenatal US diagnosis was made at a median gestational age of 22 weeks. An initial CVR higher than 1.6 was found in 16 out of 70 patients (22.8%). Fourteen out of 16 cases developed fetal complications (87%, Odds ratio 0.001, 95% CI, 7.2e-005-0.04, p less than .0001). The overall complication rate was 20% (14 out of 70 cases). The complications observed were hydrops and hydrothorax in seven cases and hydrothorax alone in seven cases. Fifty-six fetuses (80%) did not develop any complications during pregnancy.

A prenatal treatment was offered to all complicated cases. One mother underwent voluntary termination of pregnancy and one fetus was not treated for maternal refusal. In the remaining 12, TAS was placed in five (Fig. 2), steroid therapy was administered in six and alcohol injection was performed in one. 

All the antenatally treated patients and the untreated case were born alive (survival rate 100%). The five cases treated with TAS underwent a C-section, while all the other ones underwent a vaginal delivery, as per obstetrical indication.

Sixty-three fetuses (90%) were asymptomatic at birth and did not require any neonatal resuscitation. Half of the patients submitted to fetal therapy (2 TAS, 4 steroid therapy) and the untreated one presented with neonatal respiratory distress and required mechanical ventilation at birth (Fig. 1, 3). These patients, representing the 10% of the entire series (7 out of 70 cases), the 43.75% of the patients with a CVR > 1.6 (p less than 0.0001) and the 50% of the complicated patients (p less than 0.0001), required early surgery in the neonatal period, by the first month of life. Early surgery was performed through conventional thoracotomy.

**Figure:**
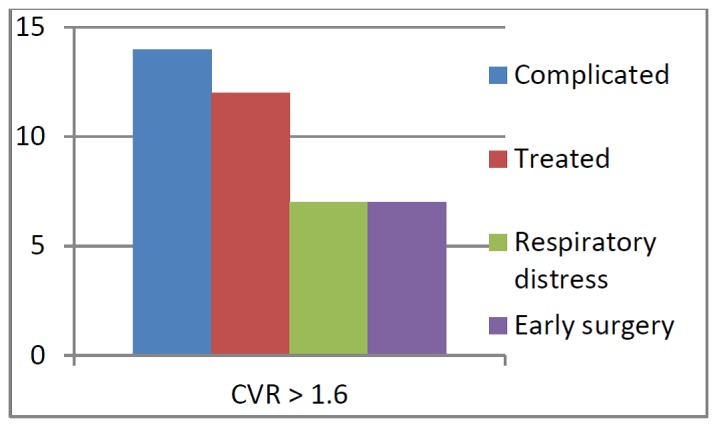
Figure 1: Outcome of patients with prenatal complications.

**Figure:**
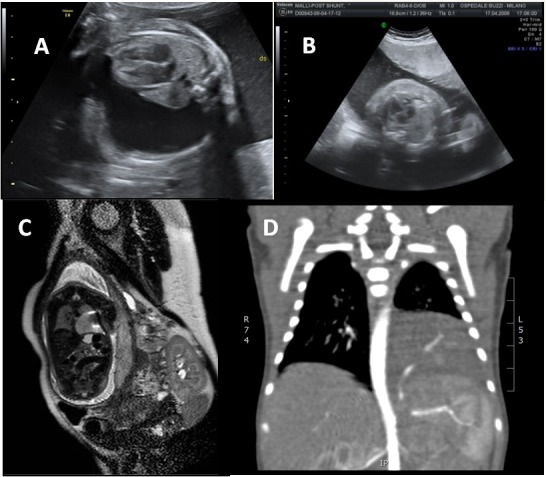
Figure 2: Case prenatally treated with thoraco-amniotic shunt positioning. Fetal US imaging of hydrothorax (A); reduction of fetal hydrothorax after TAS positioning (B: US imaging and C: fetal MRI); CT-scan at birth (D).

**Figure:**
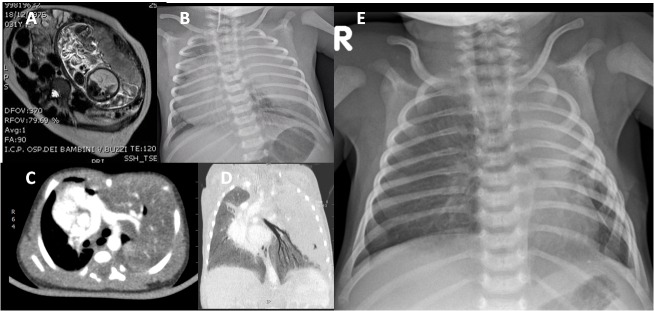
Figure 3: Large left-sided complicated lesion not submitted to fetal therapy for parental refusal: fetal MRI (A), chest X-ray (B) and CT scan (C, D) at birth; the child needed mechanical ventilation at birth and required open surgery by the first 24 hours of life (E: postoperative chest X-Ray).

In the remaining six prenatally treated patients (3 TAS, 2 steroid therapy, 1 alcohol injection), who did not require resuscitation at birth, surgery could be planned after one month of life, between three and six months. It was eventually required in five cases only, because the patient prenatally treated with alcohol injection showed a complete regression of the lesion at follow-up.

## DISCUSSION

PM represent a heterogeneous group of congenital anomalies that, although rare, can lead to a considerable morbidity and mortality. CPAM are the most common lung lesions to be identified by fetal US imaging [10]. CPAM is characterized by an overgrowth of terminal respiratory bronchioles that form cysts of various sizes [7, 11]. Given the on-going improvements in prenatal imaging, smaller CPAM can be now diagnosed with increasing frequency, so that the current incidence of CPAM is reported as 1 in 11,000-12,000 live births [3, 10]. A combination of small cyst adenomatoid malformation with an anomalous systemic blood supply is often referred to as "hybrid lesion", i.e. a cross between a form of CPAM and bronchopulmonary sequestration [12]. This is more usually discovered at postnatal surgery [2].

Prenatal US characterizes many features of PM, such as size, position, macrocystic versus microcystic appearance, evaluation of blood supply and venous drainage by Doppler US, degree of mediastinal shift, presence of hydrops, associated malformations [11]. Prenatal US diagnosis in our series was made at a median gestational age of 22 weeks. A possible bias inherent to our series is that the US prenatal diagnoses were made by different Maternal-Fetal Specialists, since our period of study is quite long (15 years). 

PM are usually unpredictable in their growth between 18 and 26 weeks of gestation. Their fastest growth usually occurs between 20 and 25 weeks, with a peak occurring at approximately 25 weeks. Then a plateau is observed usually after 28 weeks [2], with a decrease in CVR reflecting continue fetal growth [11]. Some PM can also disappear completely on US evaluation [13]. We considered the CVR at the time of diagnosis (initial CVR) for the purpose of our analyses. 

CPAM volume ratio (CVR) was initially defined in 1999 by Liechty et al. to better understand the natural history of CPAM [14] and was subsequently proposed as a prognostic tool to predict the development of fetal complications, particularly hydrops [8].

Although some authors reported a threshold value of 2.0 for CVR to be the most significant [5], we believe, in accordance with others [6, 7, 8, 10, 15], that a lower threshold is more appropriate to identify fetuses who might develop complications and thus need a closer follow-up (Table 1). 

**Figure:**
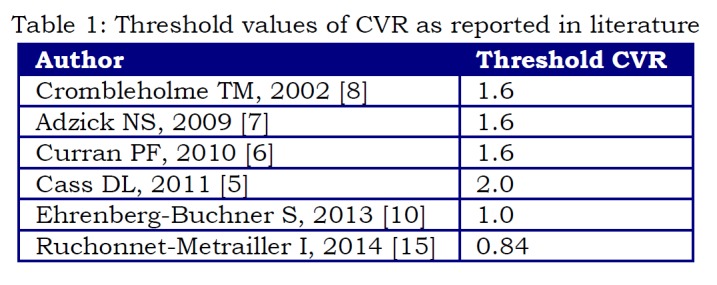
Table 1: Threshold values of CVR as reported in literature

Ruchonnet-Metrailler et al. [15] report a maximum CVR > 0.84 as an index of increased risk of respiratory complications at birth in fetuses with CPM and the most sensitive risk factor for oxygen requirement at birth. Ehrenberg-Buckner et al. [10] in their retrospective review found that a maximum CVR > 1.0 portended a 75% risk of having respiratory morbidity that required surgical resection at birth, while fetuses with a maximum CVR less than 1.0 had a probability of nearly 100% for being asymptomatic at birth. 

In our experience, an initial CVR > 1.6 was highly predictive of fetal complications (87%, p less than .0001), respiratory distress at birth and need for neonatal surgery. The occurrence of hydrops in patients with a CVR > 1.6 was 50%, in line with some literature data [16]. No one of the patients with a CVR less than 1.6 had fetal complications nor respiratory distress at birth (p less than 0.0001).

The complications observed in our series were hydrops and hydrothorax in seven cases and hydrothorax alone in further seven fetuses. The overall fetal complication rate was 20% (14 out of 70 cases). Mortality in the setting of hydrops is high and fetal intervention is warranted, depending on gestational age [3]. Proposed antenatal interventions include medical options (maternal administration of steroids) for microcystic lesions [6], intra-uterine puncture or shunting of macrocystic masses, alcohol embolization or lasering of the feeding vessel, lobectomy via hysterotomy for more solid masses and resection while on placental circulation [4, 5, 7, 16, 17]. Both puncture and TAS allow for decompression of the cysts and/or the thoracic cavity, with relief of both cardiac and pulmonary compression [3]. Lasering or injection of sclerosing agents has been described for management of microcystic lesions, where cysts are too small to be decompressed [3]. In our series, a prenatal treatment was offered to all the complicated cases, according to the indication of the Maternal-Fetal Specialist. TAS was placed in five, steroid therapy was administered in six and alcohol injection was performed in one patient. One mother underwent voluntary termination of pregnancy. One fetus with large macrocystic CPAM was not treated for maternal refusal.

Sixty-three fetuses (90%) were asymptomatic at birth and did not require any neonatal resuscitation. Seven (53.8%) out of 13 complicated cases presented with respiratory distress at birth. In these newborns (10% of the entire series), early surgery was needed, during the first days of life, because of respiratory distress and the need for mechanical ventilatory support. These patients represent the 43.75% of the patients with a CVR > 1.6 (p less than 0.0001) and the 50% of the complicated patients (p less than 0.0001), thus showing that CVR >1.6 and the presence of fetal complications can be considered as predictors of respiratory distress at birth and need for early surgery. The chosen approach in these cases was thoracotomy, since the size of the lesions and the respiratory and general conditions of the neonates made a thoracoscopic approach unsafe.

Large series report a CPAM-related perinatal mortality of around 10-15% [11]. The cause of neonatal mortality is not clear, but is suggested to be associated with lung hypoplasia [13]. We did not observe any perinatal death in our series, although we agree with the argument that neonates born with PM, particularly when complicated and submitted to fetal therapies, should be regarded by perinatologists as a high-risk population [13]. Morbidity includes both respiratory distress related to the PM and complications related to prematurity, often due to fetal interventions [13].

In asymptomatic children, our usual management comprises a chest X-ray at birth, a CT scan between three and six months o life and elective surgery between six and ten months of life. We believe, in accordance with several authors [7, 9, 16, 18], that elective resection in asymptomatic infants is advised to prevent postnatal complications, such as chest infections and sepsis – the risk of infection for congenital PM having been estimated to range between 10% to 30% within the first year of life [4], to reduce the risk of postoperative complications (compared to emergency surgery) [9, 19], to prevent malignant evolution (risk lower than 1%) [16, 20], to encourage compensatory lung growth and also to reduce the need for follow-up imaging studies. Moreover, elective surgery allows thoracoscopic resection, which can provide a shorter hospital stay, decreased post-operative pain and better cosmesis [18, 21].

## CONCLUSION

CVR greater than 1.6 appears to be a major predictor of outcome of fetal pulmonary malformations (PM). It is a useful tool to follow-up PM fetuses and to stratify their risk of complications, the need for fetal interventions and the immediate postnatal outcome. Early surgery can be needed, by the first month of life, because of respiratory distress and the need for mechanical ventilatory support. The chosen approach in these cases is thoracotomy, since the size of the lesions and the respiratory and general conditions of the neonates can make a thoracoscopic approach unsafe. The vast majority of PM are asymptomatic at birth and later in life and only a small group of fetuses, who presented with fetal complications, require prenatal and postnatal treatment and support. 

## Footnotes

**Source of Support:** None

**Conflict of Interest:** None
